# Distribution and Interaction of Murine Pulmonary Phagocytes in the Naive and Allergic Lung

**DOI:** 10.3389/fimmu.2018.01046

**Published:** 2018-05-16

**Authors:** Franziska M. Hoffmann, Johann L. Berger, Imke Lingel, Yves Laumonnier, Ian P. Lewkowich, Inken Schmudde, Peter König

**Affiliations:** ^1^Institute of Anatomy, University of Lübeck, Lübeck, Germany; ^2^Airway Research Center North (ARCN), German Center for Lung Research (DZL), Lübeck, Germany; ^3^Institute for Systemic Inflammation Research, University of Lübeck, Lübeck, Germany; ^4^Division of Immunobiology, Cincinnati Children’s Hospital Medical Center, Cincinnati, OH, United States; ^5^Department of Pediatrics, University of Cincinnati, Cincinnati, OH, United States

**Keywords:** dendritic cells, macrophages, lung, immunohistochemistry, spatial distribution, antigen uptake, allergic airway disease

## Abstract

The division of labor between pulmonary phagocytic subsets [macrophage/monocyte and dendritic cell (DC) subpopulations] has been described at the functional level. However, whether these lung phagocytes also display unique spatial distribution remains unclear. Here, to analyze cellular distribution in lung compartments and contacts between phagocyte subpopulations, we established an immunohistochemistry (IHC)-based method to clearly identify murine lung phagocyte subsets *in situ* based on differential expression of CD11c, CD11b, MHC-II, Langerin and mPDCA-1. Furthermore, we investigated subset-specific functional differences in antigen uptake and spatial changes upon allergic sensitization. Our staining allowed the distinction between alveolar macrophages (AMs), interstitial macrophage (IM) subpopulations, CD11b^+^ DC subpopulations, CD103^+^ DCs, and plasmacytoid DCs (pDCs). We identified interstitial regions between airways and around airways as regions of IM/CD11b^+^ DC/CD103^+^ DC clusters, where a subset of IMs (IM2) and CD103^+^ DCs formed intense contacts that decreased upon allergic sensitization. These data indicate functional interactions between both cell types either in steady state or after antigen encounter affecting the development of allergies or tolerance. Furthermore, we observed major antigen uptake in AMs and IMs rather than DC subpopulations that was not restricted to airways and adjacent areas. This will enable to focus future studies to immunologically relevant cellular interactions and to unravel which cells are tipping the balance between pro-inflammatory immune responses or tolerance.

## Introduction

The lung represents an interface between tissue and environment that continuously faces potential threats including inhaled airborne particles and pathogens. To prevent inflammation, lung phagocytes constantly clear inhaled particles in a silent manner (1) and secrete mediators that activate resident immune cells, typically in a tolerogenic manner (2). However, under pathological circumstances, lung phagocytes contribute to inflammatory immune responses against harmless allergens like pollen and house dust mite (HDM) feces, leading to an allergic asthma reaction instead of tolerance (3). Therefore, in order to protect tissue homeostasis, the balance between pro- and anti-inflammatory activities of lung phagocytes is tightly regulated.

Although the precise factors responsible for promoting pro- and anti-inflammatory responses remain incompletely understood, unique subsets of lung phagocytes including dendritic cells (DCs) and macrophages may preferentially act in a more pro- or anti-inflammatory manner (1, 2, 4). Subsets of pulmonary macrophages are classically defined based on localization [i.e., alveolar macrophages (AMs) versus interstitial macrophages (IMs)] and more recently based on cell function and surface marker expression into IM subsets IM1-3 (5). Regardless of localization, the low migratory potential of macrophages suggests a primary role in antigen clearance rather than induction of adaptive immune responses (6–8). In contrast, although different DC subsets have been described, they are typically more immunostimulatory owing to their more pronounced migratory capacity. For example, CD11b^+^ conventional DCs (cDCs) are regarded as inducers of Th2/Th17 immunity, while CD103^+^ cDCs are primarily involved in cross-presentation of antigens in anti-viral responses and tolerance induction (8–12). These cDC subsets are complemented by plasmacytoid DCs (pDCs), which are primarily involved in anti-viral immune responses and tolerance, and, under inflammatory conditions, by monocyte-derived DCs (moDCs) (9, 13–15). In addition, recent studies showed that also monocytes are residing in the lung and are able to take up antigens to mimic DC function in lymph nodes (16).

Although altogether these studies describe functions of all major lung phagocyte subsets, a comprehensive study considering all subsets is still lacking. This lack is aggravated by inconsistent definitions of phagocyte subsets as well as a partial overlap of subpopulations in different studies. Furthermore, previous studies completely neglected the influence of cell localization/antigen availability and cell interaction on the function of lung phagocytes as it was reported for other organs (17). In addition, yet no comprehensive study of DCs and macrophages exists that sheds light on the localization of lung phagocytes and cell contacts between different immune cell subsets.

While increasingly sophisticated multi-color flow cytometric analysis has revealed unique populations of pulmonary phagocytes, there is little information regarding the spatial-temporal localization of these cells within the lung. We assume that cell localization is strongly linked to cell function of phagocyte subsets, and thus, where these cells are located and what types of cells are in their immediate vicinity may influence their overall pro- or anti-inflammatory activity. Therefore, our study aimed to establish a method to clearly identify lung DC and macrophage subsets by immunohistochemistry (IHC) in a comprehensive manner. Furthermore, we aimed to define localization patterns of phagocyte subsets in lung tissue and investigate potential cellular interactions both under naive and inflammatory conditions.

Herein, we developed an IHC-based strategy to localize and assess phagocytic function of multiple pulmonary phagocyte populations in naive and allergen-challenged mice, including AMs, multiple subsets of IMs, CD11b^+^, and CD103^+^ DCs. Interestingly, in both steady state and after antigen encounter, we observed a clear co-localization of CD103^+^ DCs and a subset of IMs (IM2) around airways that suggests functional interactions between cell types. Moreover, we observed antigen uptake primarily in AMs and IMs, whereas DC subsets were less active. Interestingly, the uptake in the interstitium was not restricted to airway adjacent areas but also found around blood vessels. Of note, the aforementioned contacts between CD103^+^ DCs and IM2 were reduced after antigen encounter. Altogether, this study offers for the first time a comprehensive overview of identification, localization, and cell contacts between lung DC and macrophage subsets based on an IHC approach. Furthermore, it raises the question whether the direct contact of IM2 and CD103^+^ DCs is involved in tipping the balance toward pro-inflammatory immune responses, thus directly affecting allergic sensitization.

## Results

### IHC Allows Distinction of Lung Phagocyte Subsets for Localization and Quantification *In Situ*

**Figure 1:**
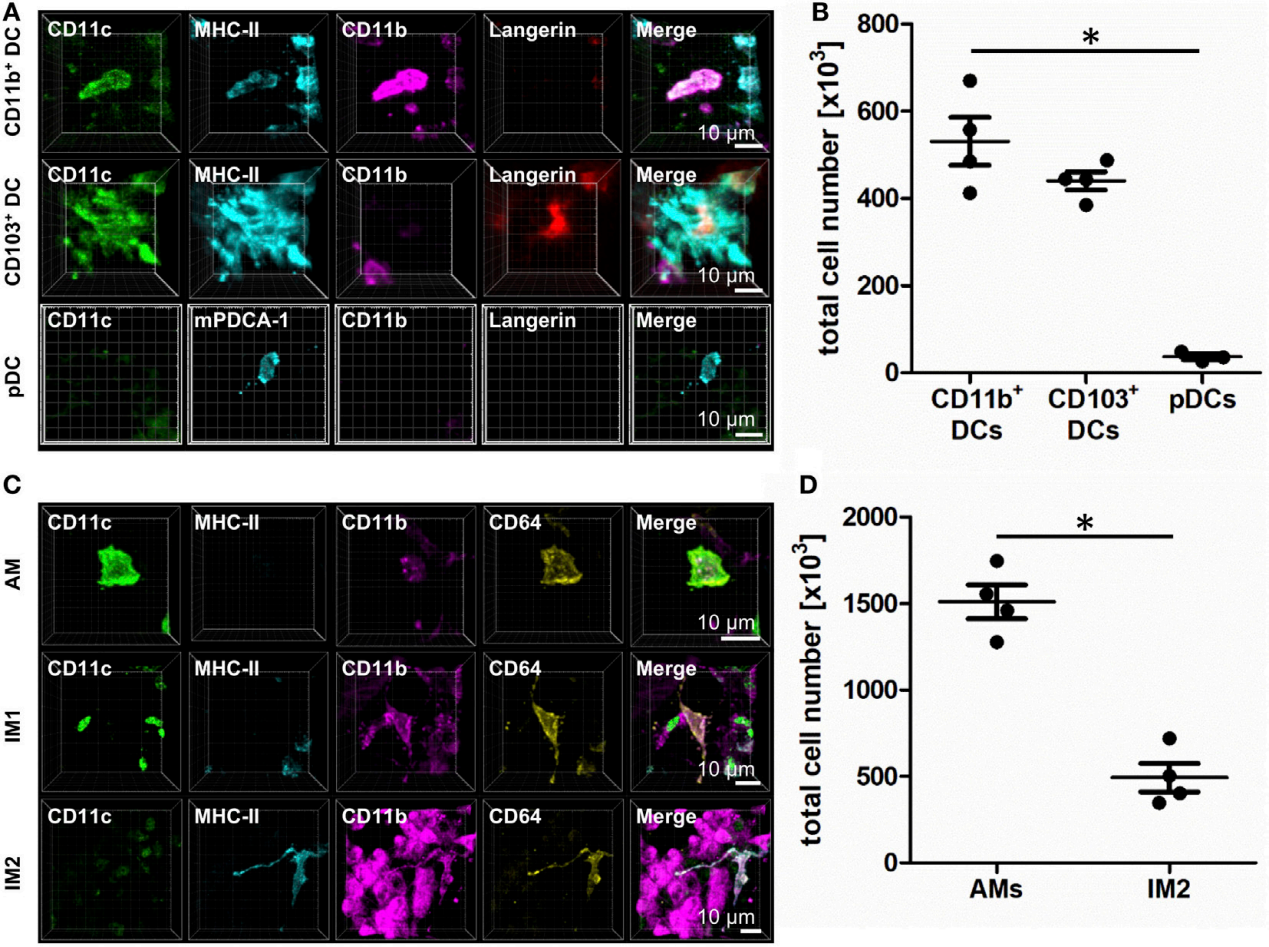
Identification of lung phagocyte subsets by immunohistochemistry (IHC). Precision cut lung slices (PCLS) (300 µm) from naive C57BL/6 mice were generated and stained with anti-CD11c, anti-MHC-II or anti-mPDCA-1, anti-CD11b, and anti-Langerin **(A,B)** or anti-CD64 ABs **(C,D)**. Stained slices were evaluated with confocal microscopy. IHC of CD11b^+^ dendritic cells (DCs), CD103^+^ DCs, and plasmacytoid DCs (pDCs) **(A)** or alveolar macrophages (AMs), interstitial macrophages (IM)1, and IM2 **(C)**. Single-color and merged color display with CD11c (green), MHC-II or mPDCA-1 (turquois), CD11b (purple), and Langerin (red) or CD64 (yellow). Data are representative of at least three independent experiments. Quantification of CD11b^+^ DCs, CD103^+^ DCs, and pDCs **(B)** and AMs and IM2 **(D)** in total lungs. Lines indicate mean ± SEM. Differences between groups were tested by Kruskal–Wallis test (b) or Mann–Whitney *U*-test (d) for significance; *p < 0.05.

Based on the expression of CD11c, CD11b, MHC-II, Langerin, mPDCA-1, CD64, and autofluorescence, we were able to establish IHC panels that enable differentiation of CD103^+^ cDCs, CD11b^+^ cDCs, pDCs, AMs, and two unique populations of IMs termed IM1 and IM2 according to Gibbings et al. (Figure 1) (5). Langerin was used as a surrogate marker of CD103 since IHC with anti-CD103 strongly depended on fixation. In contrast to CD103, Langerin is expressed intra-cellularly but exclusively in CD103^+^ cells (Figure S1A in Supplementary Material). Therefore, to avoid confusion with existing DC definitions, Langerin^+^ cells are termed CD103^+^ DCs in the following.

CD11b^+^ cells expressed CD11c, CD11b, and MHC-II, lacked Langerin expression, and differentially expressed CD64 based on their ontogenetic background from DC or monocytic precursors (Figure 1A; Figure S1B in Supplementary Material). In naive animals, they represent about 500,000 cells per lung (Figure 1B). In contrast, CD103^+^ DCs expressed CD11c, Langerin, and MHC-II and lacked CD11b and CD64 expressions (Figure 1A; Figure S1B in Supplementary Material). In the steady state, CD103^+^ DCs totaled ~450,000 cells per lung (Figure 1B). The minority of lung DCs represent pDCs that were characterized by the expression of mPDCA-1. The expression of CD11c and CD11b failed to reach the detection limit of IHC (Figure 1A). Their numbers in naive lungs were very few—averaging ~50,000 per naive lung (Figure 1B).

Alveolar macrophages expressed CD11c and CD64 but lacked MHC-II and Langerin expressions. Furthermore, they were strongly autofluorescent in the UV channel we used to detect CD11b (Figure 1C; Figure S1C in Supplementary Material). The majority of lung phagocytes represented AMs with a total cell number of about 1,500,000 cells per lung (Figure 1D). IM1 were identified as cells expressing CD11b and CD64 but stained negatively for CD11c, Langerin and MHC-II (Figure 1C). Lastly, IM2 were characterized by the expression of CD11b, MHC-II, and CD64 and a lack of CD11c and Langerin (Figure 1C; Figure S1C in Supplementary Material). The abundance of IM2 was comparable to CD11b^+^ and CD103^+^ DCs, averaging ~500,000 cells per naive mouse (Figure 1D).

**Figure 2:**
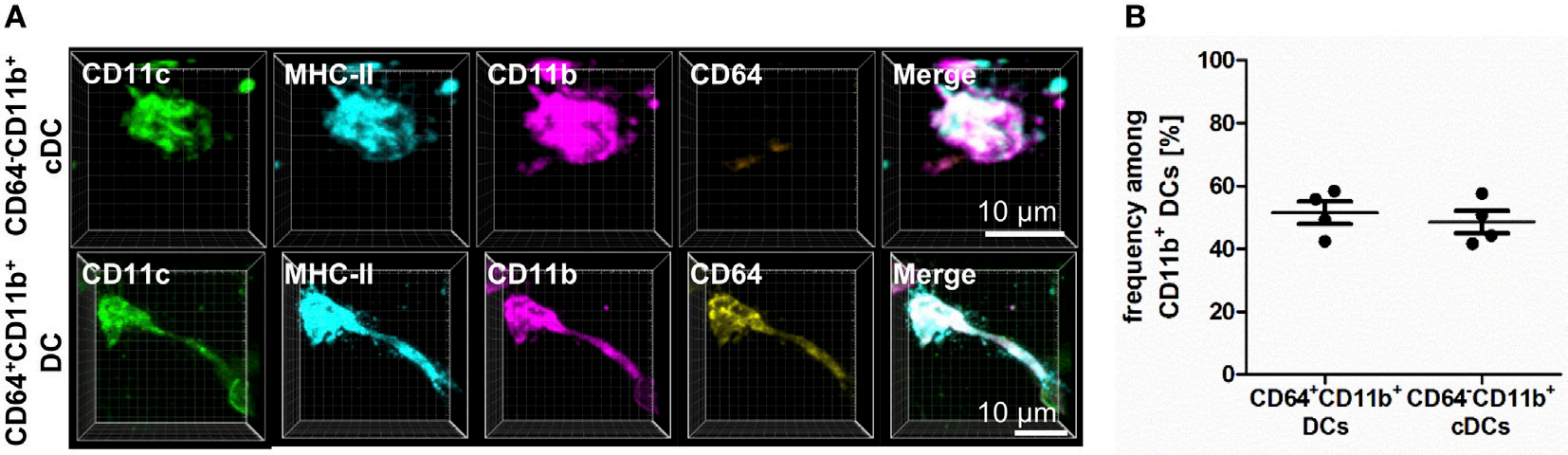
CD11b^+^ dendritic cells (DCs) differ in CD64 expression based on their ontogenetic origin. Precision cut lung slices (PCLS) (300 µm) from naive C57BL/6 mice were generated and stained with anti-CD11c, anti-MHC-II, anti-CD11b, and anti-CD64 mABs. Stained slices were evaluated with confocal microscopy. **(A)** Immunohistochemistry (IHC) of CD11b^+^ conventional DCs (cDCs) and CD64^+^CD11b^+^ DCs. Single-color and merged color display with CD11c (green), MHC-II (turquois), CD11b (purple), and CD64 (yellow). Data are representative of four independent experiments. **(B)** Frequency of CD11b^+^ cDCs and monocyte-derived DCs (moDCs) among CD11b^+^ DCs. Lines indicate mean ± SEM. Differences between groups were tested by Mann–Whitney *U*-test **(B)** for significance.

In addition to the characterization of AMs and IMs, the usage of CD64 enabled us to identify two major populations of CD11b^+^ DCs—those originating from a DC precursor (CD64^−^) or those from monocytic origin (CD64^+^) (Figure 2). Although both subpopulations equally contributed to the CD11b^+^ compartment and shared the expression of CD11c, CD11b, and MHC-II, they clearly differed in CD64 expression (Figure 2). While CD11b^+^ and CD103^+^ DC could be readily identified using flow cytometric techniques, the overall pattern of CD64 expression differed markedly in flow- versus IHC-based methods. While IHC suggested CD64 was expressed on ~50% of all CD11b^+^ DCs, only ~5% of CD11b^+^ DCs expressed CD64 via flow. Of note, C5aR1 showed an exact overlap with CD64 expression in IHC-based techniques, and the observation that ~50% of CD11b^+^ DCs expressed C5aR1 by flow (Figure S2 in Supplementary Material) suggests that C5aR1 could serve as a more reliable marker to identify this DC population by different approaches. Thus, we were able to specifically identify lung phagocyte subsets via IHC for further localization and functional analyses.

### Lung Phagocyte Subsets Exhibit Specific Distribution Patterns *In Situ*

We next investigated the localization of the phagocyte subpopulations *in situ*. Additional use of antibodies to alpha-smooth muscle actin (α-SMA) (18) and autofluorescence allowed us to specifically identify airways, pulmonary arteries, intra-acinar arteries, alveolar ducts, and veins (Figure S3 in Supplementary Material). Airways were characterized by highly autofluorescent airway epithelial cells (Figure S3 in Supplementary Material, top row). Close to airways, pulmonary arteries are located and characterized by a bright, confluent, homogenous autofluorescence (Figure S3 in Supplementary Material, top row). Intra-acinar arteries exhibited a similar fluorescence pattern with a lower intensity due to a smaller diameter (Figure S3 in Supplementary Material, middle row). Each intra-acinar artery was surrounded by up to four alveolar ducts, which appeared as black holes at both sides of the vessel (Figure S3 in Supplementary Material, middle row). Finally, veins showed an irregular autofluorescence pattern and no proximity to alveolar ducts (Figure S3 in Supplementary Material, bottom row). As autofluorescence alone was sufficient to identify these structural features of the lung, this parameter alone was used to further investigate the localization of lung phagocytes.

**Figure 3:**
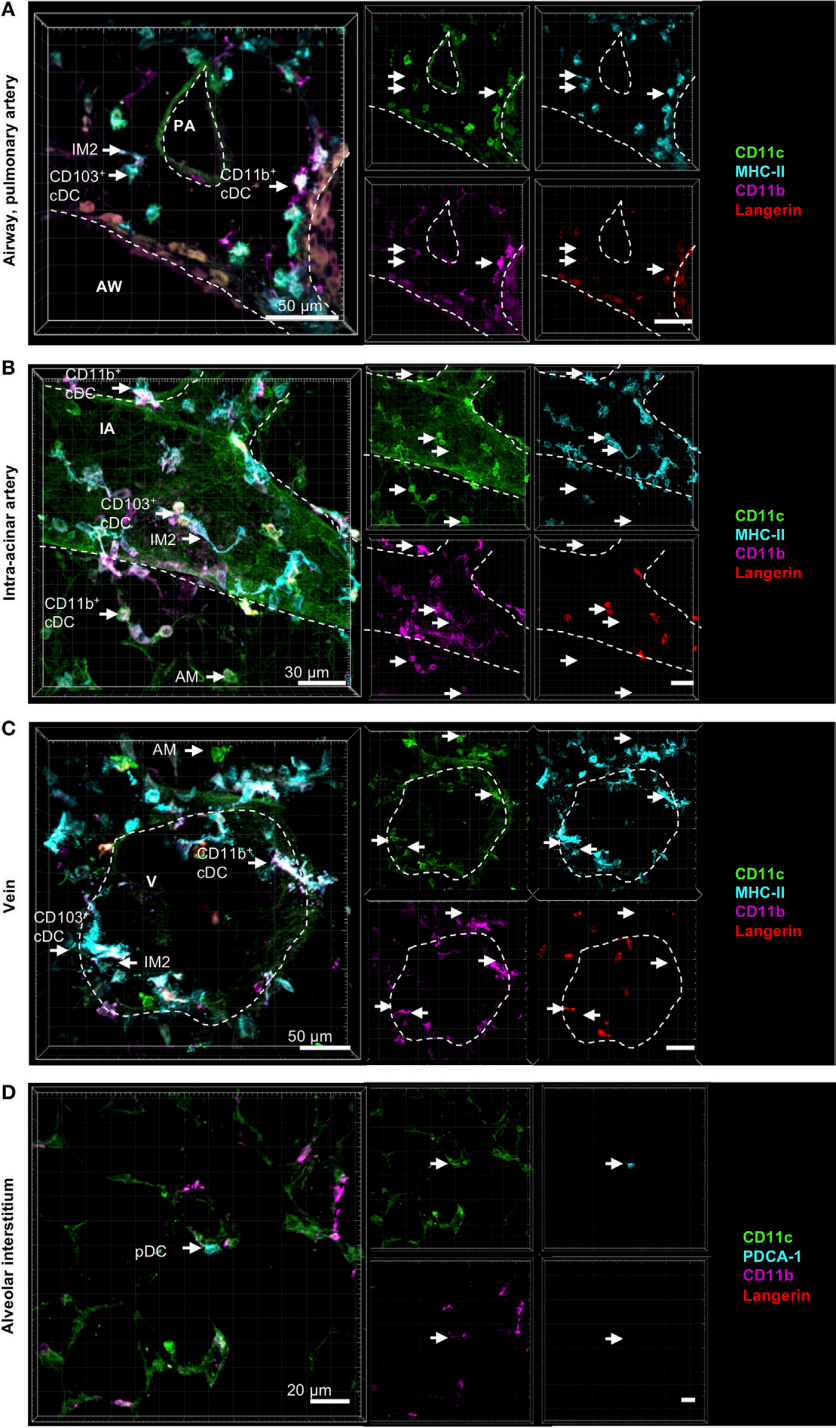
Localization of pulmonary dendritic cell (DC) and macrophage subsets by immunohistochemistry (IHC). Precision cut lung slices (PCLS) (300 µm) from naive C57BL/6 mice were generated and stained with anti-CD11c (green), anti-MHC-II or mPDCA-1 (turquois), anti-CD11b (purple), and anti-Langerin (red) ABs. Stained slices were evaluated with confocal microscopy. Conventional DCs (cDCs) and interstitial macrophages (IMs) were localized in the interstitium around AWs and pulmonary arteries **(A)**, around IAs **(B)**, and Vs **(C)**, while alveolar macrophages (AMs) were only located in the alveolar lumen **(A–C)**. Plasmacytoid DCs (pDCs) were primarily located in the alveolar interstitium **(D)**. Dashed lines indicate AWs and/or vessels. Abbreviations: AW, airway; PA, pulmonary artery; V, vein; IA, intra-acinar artery. Data are representative of at least three independent experiments.

In the resting lung, both CD11b^+^ and CD103^+^ DCs were distributed in the airway interstitium close to the airway epithelium in the connective tissue between airway and pulmonary artery (Figure 3A), around intra-acinar arteries (Figure 3B) and around veins (Figure 3C). However, neither CD11b^+^ nor CD103^+^ DCs were integrated into the epithelium nor found to send dendrites into the airway lumen penetrating the epithelial barrier. pDCs were widely distributed across the alveolar interstitium close to blood vessels but not close to the airway epithelium (Figure 3D).

**Figure 4:**
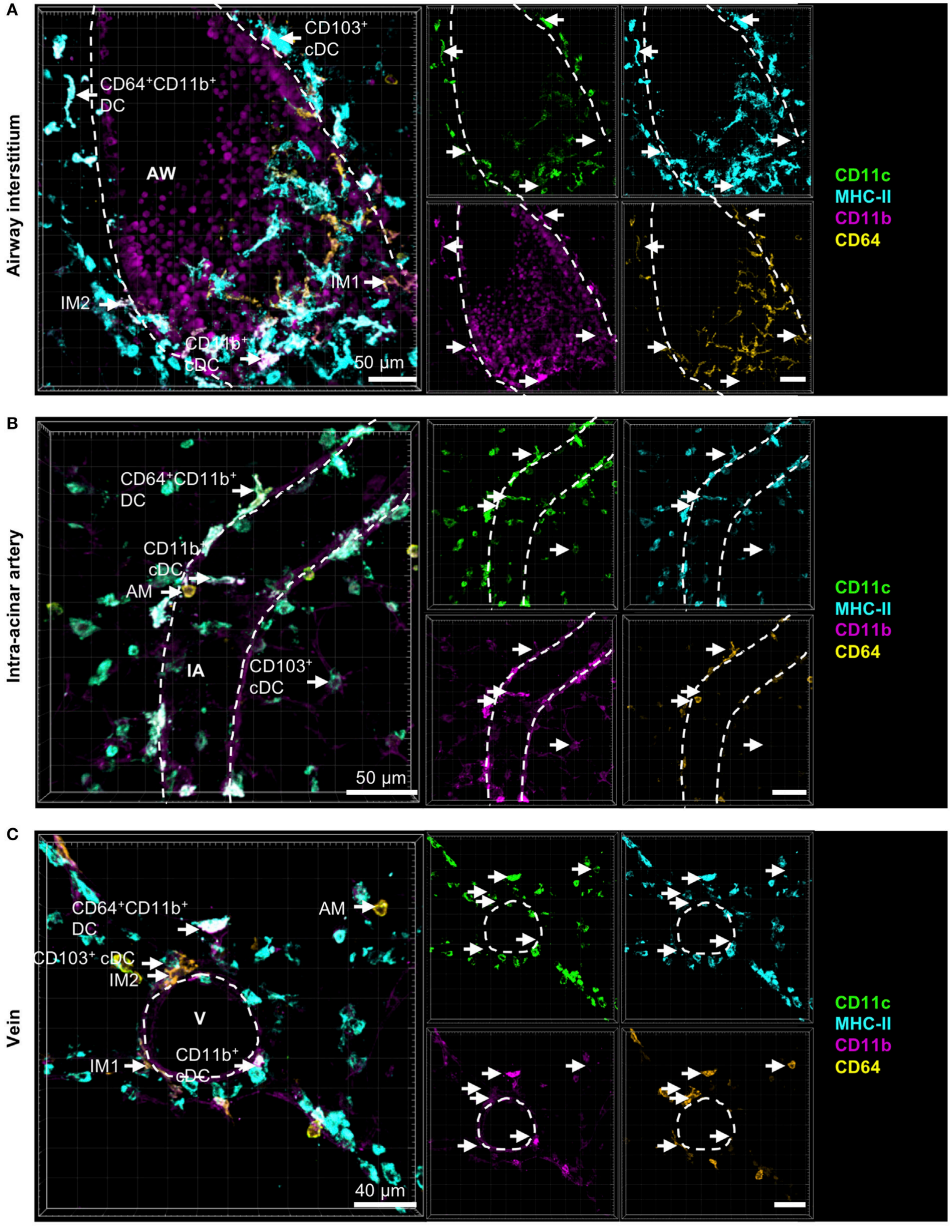
Cells with monocytic origin are located around AWs and blood vessels. Precision cut lung slices (PCLS) (300 µm) from naive C57BL/6 mice were generated and stained with anti-CD11c (green), anti-MHC-II (turquois), anti-CD11b (purple), and anti-CD64 (yellow) mABs. Stained slices were evaluated with confocal microscopy. Cells with monocytic origin were observed in the interstitium around AWs **(A)**, IAs **(B)**, Vs **(C)**, and in the alveolar lumen **(A–C)**. Dashed lines indicate structures of AWs and vessels. Abbreviations: AW, airway; V, vein; IA, intra-acinar artery. Data are representative of at least three independent experiments.

Comparable to CD11b^+^ and CD103^+^ DCs, IM2 were located in the bronchial interstitium in proximity to blood vessels (Figures 4A,C). As expected, AMs were located in the alveolar lumen, often they were observed in close proximity to the alveolar epithelium suggesting that they are attached to it (Figures 4B,C). As approximately 50% of all CD11b^+^ DCs are of monocytic origin (CD64^+^), we also compared the localization of tissue-resident IM1 and other monocyte-derived lung phagocytes. Both CD64^+^ DCs and CD64^−^ DCs have the same distribution pattern around airways and blood vessels. Surprisingly, IM1 were mainly located in the airway interstitium close to the airway epithelium and only rarely around blood vessels (Figures 4A,C). In summary, we were able to identify lung phagocyte subsets from the DC and macrophage compartment in the lung tissue. Furthermore, autofluorescence of the lung tissue enabled us to orientate *in situ* and to observe distributional differences of the investigated phagocyte subsets in the lung tissue without the need of further antibody staining of lung structures.

### IM1, IM2, and CD11b^+^ DCs Serve as Major Antigen-Uptaking Cells in Lung Tissue

**Figure 5:**
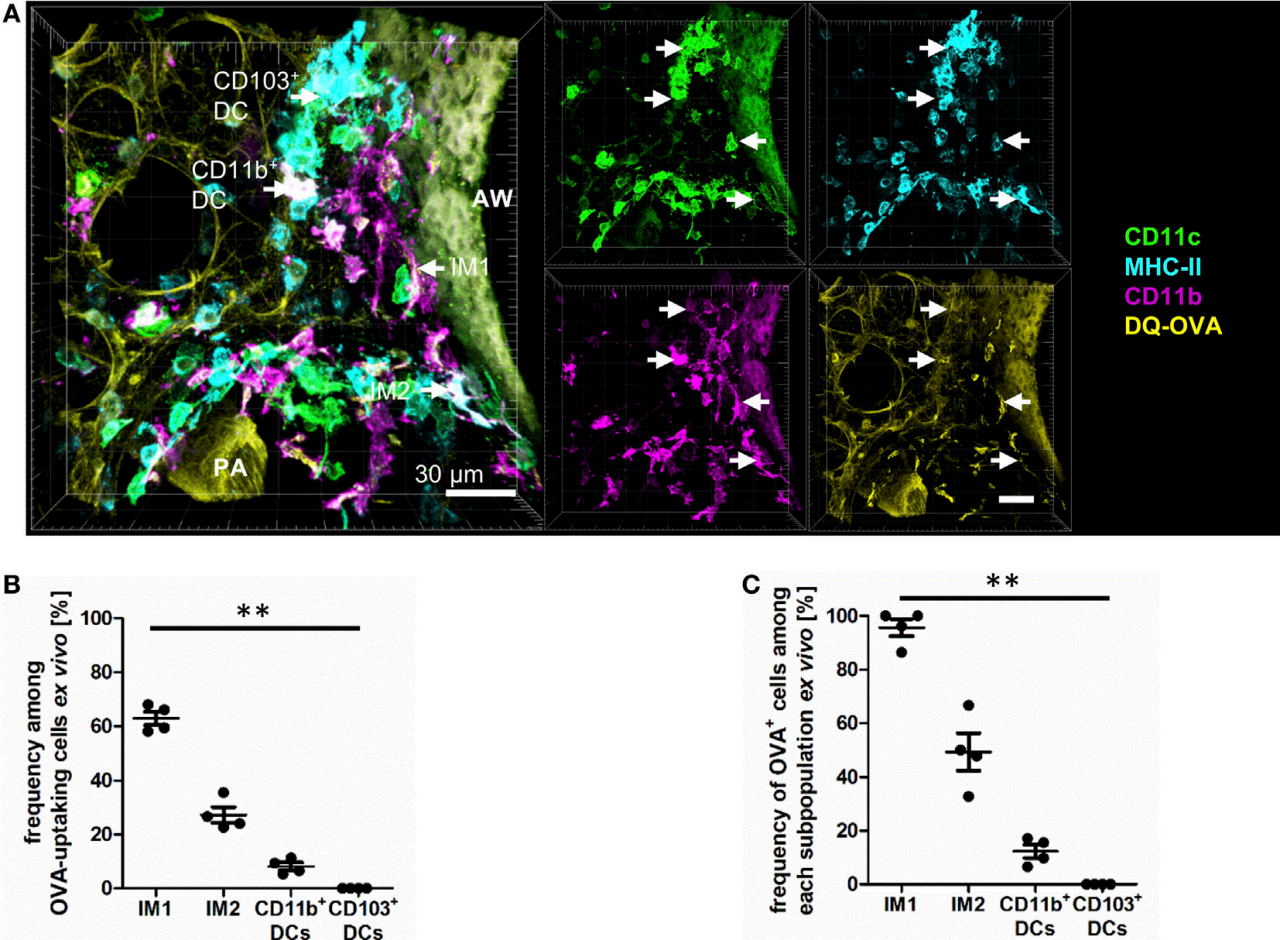
Interstitial macrophages (IM)1 and IM2 are major antigen-uptaking cells in the lung *in vitro*. Precision cut lung slices (PCLS) (300 µm) from naive C57BL/6 mice were generated and incubated *in vitro* with 100 µg house dust mite extract (HDM) mixed with 40 µg DQ-ovalbumin (OVA). After 30 min of incubation, PCLS were fixed and stained with anti-CD11c, anti-MHC-II, and anti-CD11b mABs. Stained slices were evaluated with confocal microscopy. Fluorescence of DQ-OVA was measured in the FITC-channel. **(A)** Single-color and merged color display with CD11c (orange), MHC-II (red), CD11b (blue), and DQ-OVA (green). Data are representative of four independent experiments. **(B)** Frequency of IM1, IM2, CD11b^+^ conventional DCs (cDCs), and CD103^+^ dendritic cell (DCs) among OVA-uptaking cells. **(C)** Frequency of DQ-OVA^+^ cells within each phagocyte subset. Lines indicate mean ± SEM. Differences between groups were tested by Kruskal–Wallis test **(B,C)** for significance; **p < 0.01.

To next examine the phagocytic capacity of each pulmonary phagocyte, we prepared viable lung slices from naive mice and incubated them for 30 min *in vitro* with a mixture of HDM (to induce a pro-inflammatory milieu) and DQ-ovalbumin (OVA) (to track antigen uptake). DQ-OVA is characterized by a strong fluorescence in the FITC-channel after uptake and antigen processing. In the alveoli, antigen uptake was restricted to AMs (data not shown). While IM1 represented the most phagocytically active cell in the lung interstitium, IM2 and CD11b^+^ DCs demonstrated some phagocytic capacity (Figures 5A–C). CD103^+^ DCs did not demonstrate appreciable antigen uptake (Figures 5A–C). In general, antigen-loaded cells were typically found around the airways and blood vessels. We never observed antigen-bearing cells within the epithelial layer and did not observe protrusions through the airway epithelium.

**Figure 6:**
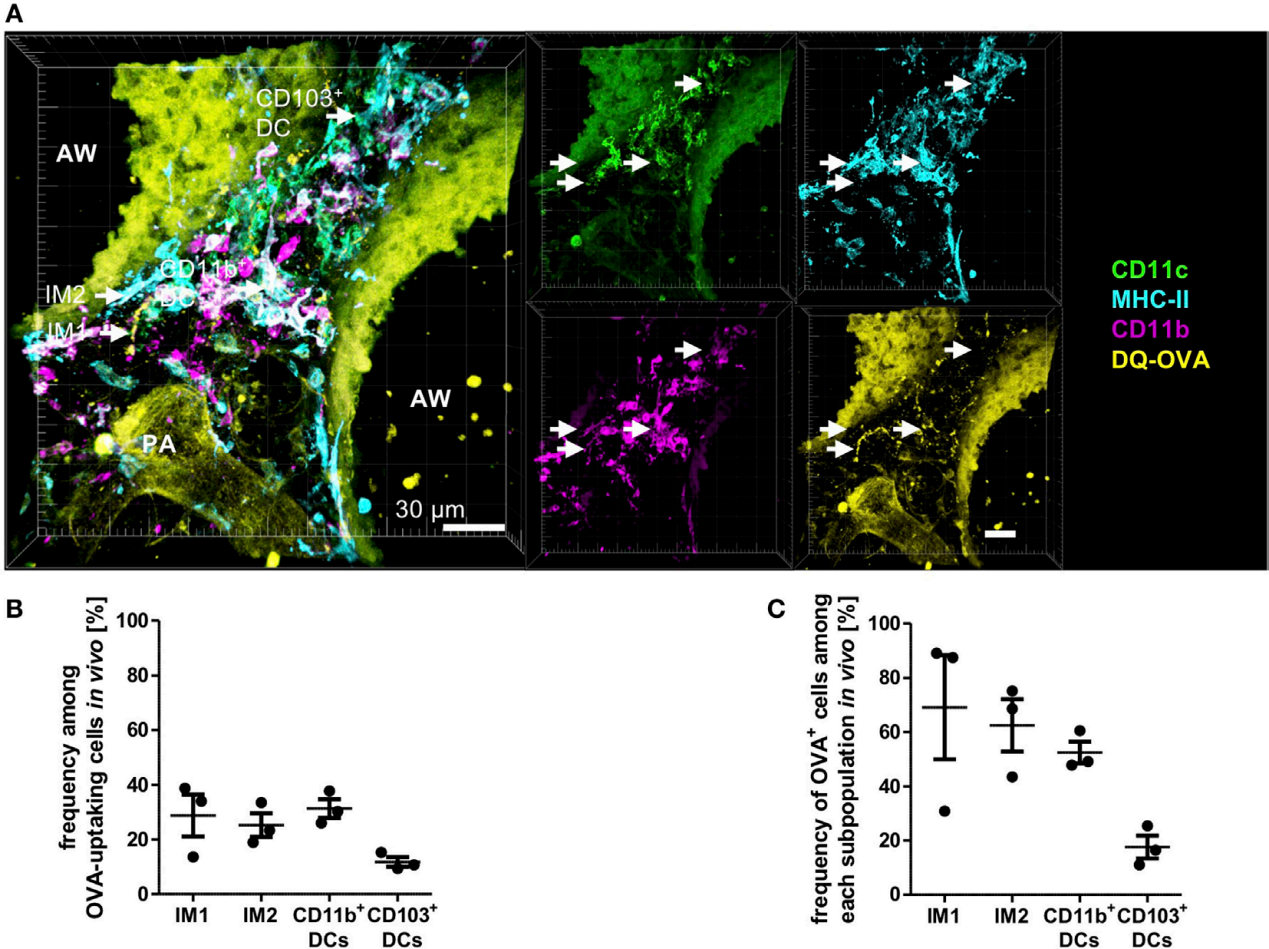
Interstitial macrophages (IM)1, IM2, and CD11b^+^ dendritic cells (DCs) equally contribute to antigen uptake in the lung *in vivo*. C57BL/6 mice were anesthetized and immunized intratracheally with 100 µg house dust mite extract (HDM) mixed with 40 µg DQ-ovalbumin (OVA). Four hours after immunization, mice were sacrificed, and precision cut lung slices (PCLS) (300 µm) were generated and stained with anti-CD11c, anti-MHC-II, and anti-CD11b mABs. Stained slices were evaluated with confocal microscopy. Fluorescence of DQ-OVA was measured in the FITC-channel. **(A)** Single-color and merged color display with CD11c (orange), MHC-II (red), CD11b (blue), and DQ-OVA (green). Data are representative of four independent experiments. **(B)** Frequency of IM1, IM2, CD11b^+^ conventional DCs (cDCs), and CD103^+^ DCs among OVA-uptaking cells. **(C)** Frequency of DQ-OVA^+^ cells within each phagocyte subset. Lines indicate mean ± SEM. Differences between groups were tested by Kruskal–Wallis test **(B,C)** for significance.

The method of *in vitro* antigen uptake allowed us to offer antigen in excess bypassing the physiological epithelial barrier generating equal antigen access to all phagocyte subsets. *In vivo* antigen access is limited by the epithelial barrier, and the proximity of phagocytes subsets to the entrance routes of antigen into the lung tissue. Therefore, a second set of experiments was performed offering antigen *in vivo* via the intratracheal route. Mice were anesthetized and immunized with a mixture of HDM and OVA, and antigen uptake was determined 4 h later by IHC. In this more physiologic setting, we observed equal contribution of IM1, IM2, and CD11b^+^ DCs to antigen uptake (Figures 6A,B). Interestingly, in contrast to the *in vitro* approach, CD103^+^ DCs were readily able to take up and process antigen albeit to a lower extent than the other populations. This was reflected by the percentage of OVA^+^ cells within the subpopulations. While 60–70% of IM1, IM2, and CD11b^+^ DCs took up and processed antigen, only 20% of CD103^+^ DCs were able to take up DQ-OVA (Figure 6C). Antigen uptake was located both in the connective tissue around airways and distant from airways in the lung parenchyma around arteries and veins. Furthermore, antigen uptake could be observed in the alveolar space by AMs (data not shown). In conclusion, we determined AMs, IM1, IM2, and CD11b^+^ DCs as major antigen-uptaking cells *in vitro* and *in vivo*.

### During Allergic Sensitization CD11b^+^ DCs Are Recruited to the Lung and Differentiate from IM1

**Figure 7:**
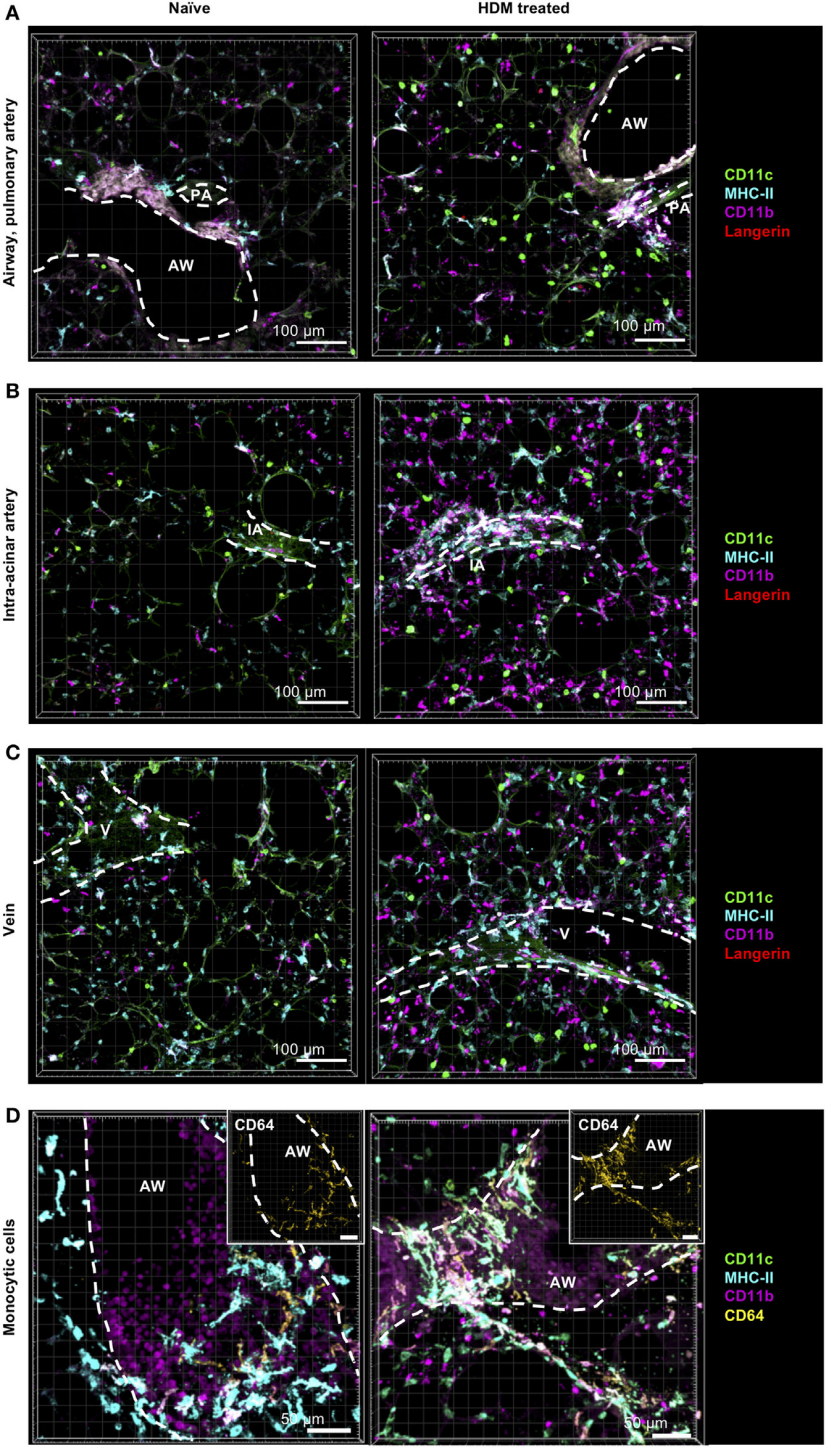
Allergic sensitization induces cellular accumulation of CD11b^+^ monocyte-derived dendritic cells (moDCs) around AWs and blood vessels. C57BL/6 mice were anesthetized and immunized intratracheally with house dust mite extract (HDM) (100 µg). A total of 24 h after immunization mice were sacrificed, precision cut lung slices (PCLS) (300 µm) were generated and stained with anti-CD11c (green), anti-MHC-II (turquois), anti-CD11b (purple), and anti-Langerin (red) or anti-CD64 (yellow) ABs (right panel). Naive C57/BL6 mice served as controls (left panel). Stained slices were evaluated with confocal microscopy. Localization of conventional DCs (cDCs), interstitial macrophages (IMs), and alveolar macrophages (AMs) was determined in the interstitium around AWs and pulmonary arteries **(A)**, around IAs **(B)**, Vs **(C)** and in the alveolar lumen **(A–C)**. Accumulation of CD64^+^CD11b^+^ DCs was determined around AWs **(D)**. Dashed lines indicate AWs and/or vessels. Abbreviations: AW, airway; V, vein; IA, intra-acinar artery. Data are representative of at least three independent experiments.

Since it is known that inflammatory cells are recruited rapidly to the site of inflammation, we next aimed at determining the numbers of pulmonary DCs and macrophages after allergen challenge compared to steady-state conditions. Mice were treated with a single dose of HDM, and 24 h later, cellular composition and localization in the lung were determined. As expected, we observed a general cellular influx upon HDM immunization, although localization of DCs and macrophages was largely unaffected (Figures 7A–D). This increase was mainly due to an increase in CD11b^+^ DCs and was not restricted to specific areas but seen around airways, pulmonary arteries (Figure 7A), intra-acinar arteries (Figure 7B), and veins (Figure 7C) where formation of cell aggregates could be observed. More specifically, the increase in CD11b^+^ DCs was associated with an increased frequency of CD64^+^CD11b^+^ DCs (up to ~85%), suggesting increased number of CD11b^+^ cDCs of monocytic origin (Figure S4 in Supplementary Material). Interestingly, we also detected an increase in small CD11b^+^ cells mainly in the alveolar region and close to airways. So far, we hypothesize that these cells comprise neutrophils or eosinophils being recruited to the site of inflammation (Figures 7A–C). Furthermore, we detected an increase in IM1 that formed dense networks around blood vessels and in the alveolar lumen (Figure 7D). No considerable increase in other monocytic cells was observed around blood vessels or in the alveolar interstitium. Together, allergic sensitization led to an increase in CD11b^+^ DCs with monocytic origin and recruitment of IM1.

### IM2 and CD103^+^ DCs Form Specific Contacts Around Airways

**Figure 8:**
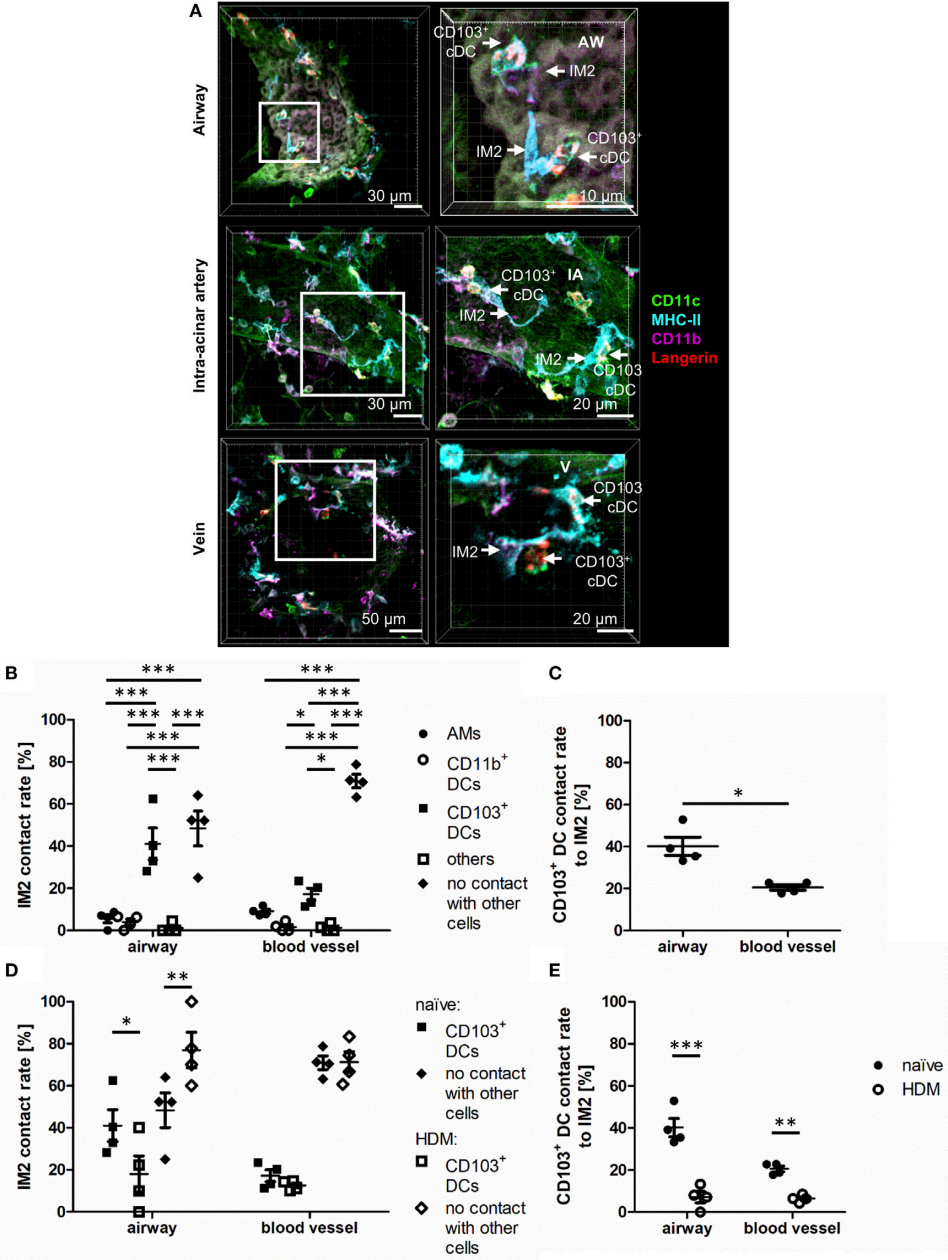
Interstitial macrophages (IM)2 and CD103^+^ dendritic cells (DCs) form specific contacts around AWs. Naive C57BL/6 mice were anesthetized and immunized intratracheally with house dust mite extract (HDM) (100 µg). A total of 24 h after immunization, mice were sacrificed, precision cut lung slices (PCLS) (300 µm) were generated and stained with anti-CD11c (green), anti-MHC-II (turquois), anti-CD11b (purple), and anti-Langerin (red) ABs. Naive C57/BL6 mice served as controls **(A)**. Stained slices were evaluated with confocal microscopy. Contact rates at the AW epithelium and blood vessels of naive mice were determined between IM2 and other phagocytes **(B)** as well as between CD103^+^ DCs and IM2 **(C)**. Furthermore, the contact rates after HDM immunization were assessed at the AW epithelium and blood vessels between IM2 and CD103^+^ DCs **(D)** and CD103^+^ DCs and IM2 **(E)**. Abbreviations: AW, airway; IA, intra-acinar artery; V, vein. Data are representative of at least four independent experiments. Lines indicate mean ± SEM. Differences between groups were tested by two-way ANOVA **(B,D,E)** or Mann–Whitney *U*-test **(C)** for significance; *p < 0.05, **p < 0.01, ***p < 0.001.

An IHC-based identification approach allows the examination of possible cell interactions between pulmonary phagocytes. During the previous experiments, it became evident that around airways and blood vessels CD103^+^ DCs were often in direct contact to IM2 (Figure 8A). In order to quantify this observation, we compared interactions between IM2 and other lung phagocytes confirming that ~40% of airway adjacent IM2 were in direct contact to CD103^+^ DCs (Figure 8B). An equal proportion of IM2 was in no direct contact to any other labeled cell. Contacts to AMs, CD11b^+^ DCs or other cells played only a minor role in a range of less than 10% of all IM2. Although we observed a slightly decreased frequency of IM2 in contact with CD103^+^ DCs at locales more distant from the airways, this was not associated with increased interactions with other labeled lung cells but rather to an increase in IM2 with no direct interaction partner. Analyzing the interaction between IM2 and CD103^+^ DCs *vice versa* revealed that 20–40% of all CD103^+^ DCs were in direct contact with IM2 around blood vessels and airways, respectively (Figure 8C).

The observation of this potential site of interaction between IM2 and CD103^+^ DCs led us to the question whether this might have a functional impact during allergic immunization. Therefore, we analyzed changes in contact rates after immunization with HDM. Interestingly, around airways, allergic immunization led to reduced contacts between IM2 and CD103^+^ DCs. This observation was valid for both interaction rates between IM2 and CD103^+^ DCs (Figure 8D) and *vice versa* (Figure 8E). Around blood vessels, this down-regulation was less pronounced compared to regions around airways. In summary, we observed an increased contact rate between IM2 and CD103^+^ DCs that was reduced upon HDM immunization suggesting a functional cross-talk between both cell types in steady state.

## Discussion

In this study, we established an IHC staining panel that allowed us for the first time to comprehensively identify pulmonary macrophage and DC subsets by simultaneous staining. According to current literature, pulmonary DCs comprise CD11b^+^ DCs, CD103^+^ DCs, and pDCs. While CD103^+^ DCs and pDCs are generally considered homogenous populations, CD11b^+^ DCs can be further subdivided into subpopulations based on ontogenetic differences, as they have been shown to arise from either distinct DC precursors, or monocytes (so-called moDCs). Within CD11b^+^ DCs, we observed two distinct populations based on the expression of CD64. We speculate that these populations represent the two ontogenetic sources of these CD11b^+^ DCs—those derived from true DC precursors (CD64^−^) versus those arising from monocytes (CD64^+^). Our observations that the frequency of CD64^+^ CD11b^+^ DCs can range from 5% (flow-based detection) to 50% (IHC-based detection) are consistent with a wide range of frequencies observed in previous publications (9, 19, 20). Interestingly, our study also showed that CD64 is always co-expressed with C5aR1 in IHC and that C5aR1 is expressed in almost 50% of all CD11b^+^ DCs by flow cytometry. As C5aR1 is expressed in all cells of monocytic origin (20, 21), we argue that C5aR1 may serve as a more appropriate marker for the identification of true moDCs in flow-based techniques, as has been proposed elsewhere (19).

These lung phagocytes are complemented by macrophage subpopulations, which are subdivided into IMs and AMs, based largely on localization within the lung interstitium versus alveolar spaces, respectively. AM subpopulations differ in the behavior of cells upon experimental isolation. Based on the strength of attachment and cellular communication with epithelial cells, two subpopulations can be distinguished (4). We were unable to identify novel subsets of AMs, but our protocol identified clearly alveolar-localized cells with markers comparable to those previously described for AMs (5, 22). Only recently further subdivisions have been proposed within the IM population. Gibbings et al. published that also IMs can be distinguished into three different subpopulations (IM1, IM2, and IM3) that are defined by the expression of MerTK, CD64, CD11b, and CX_3_CR1 (5). Based on similarities in surface marker expression between our IM1 (CD64^+^CD11b^+^CD11c^−^MHC-II^−^) and those of Gibbings et al. (MerTK^+^CD64^+^CD11b^+^CD11c^−^MHC-II^low^) and our IM2 (CD11b^+^CD11c^−^MHC-II^+^) and those of Gibbings et al. (MerTK^+^CD64^+^CD11b^+^CD11c^-^MHC-II^+^), we believe that IM1 and IM2 described by our group and that of Gibbings et al. are identical (5). While we did not identify a third IM population, IM3 defined by Gibbings et al. (MerTK^+^CD64^+^CD11b^+^CD11c^+^MHC-II^+^CCR2^+^) are phenotypically similar to moDCs as defined by Plantinga et al. (CD11c^+^CD11b^+^CD64^+^MAR-1^+^MHC-II^+^CCR2^+^) (5, 9). Thus, we speculate that the “IM3” described by Gibbings et al. and the “moDCs” described by Plantinga et al. are indeed the same cell. However, based on our collective knowledge of differences of CD64^+^ and CD64^−^ expressions on cells in terms of recruitment upon inflammation, ontogenetic background, and migratory behavior upon activation, we speculate that IM3 truly represent a macrophage cell population rather than true moDCs.

**Figure 9:**
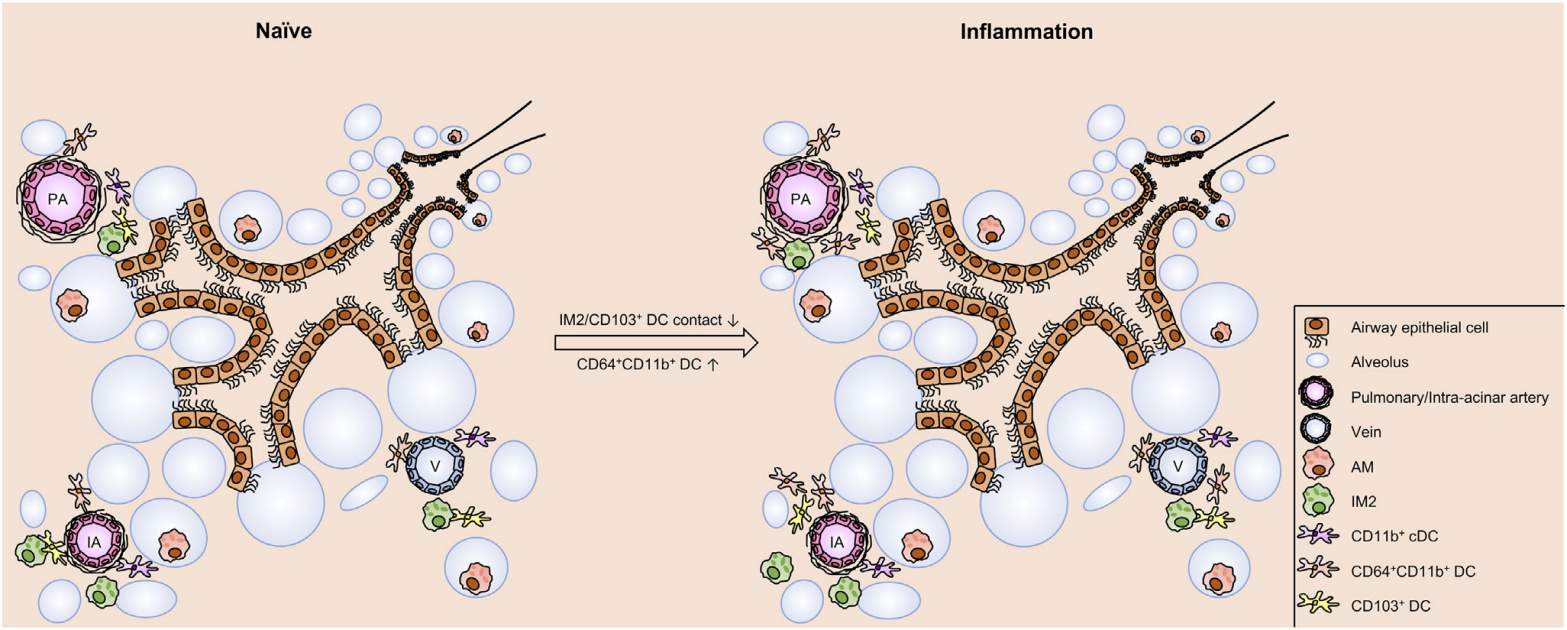
Schematic view on cell distribution and interaction in naive mice and upon allergic sensitization. In the naive lung of C57BL/6 mice clusters of phagocytes are found around IAs, pulmonary arteries, and Vs. These clusters comprise dendritic cells (DCs) (CD103^+^ DCs, CD11b^+^ DCs, and CD64^+^CD11b^+^ DCs) and interstitial macrophages 2 (IM2). Within the clusters contacts between CD103^+^, DCs and IM2 were observed. Allergic sensitization induces an increase in CD64^+^CD11b^+^ DCs without changing the distribution pattern of the investigated cell types. However, allergic immunization reduces the frequency of interactions between CD103^+^ DCs and IM2. In contrast to the aforementioned cells, alveolar macrophages (AMs) are localized both under naive and asthmatic conditions in the alveolar lumen without any close contact to other phagocytes. Abbreviations: AW, airway; IA, intra-acinar artery; V, vein.

The greatest strength of our study is the ability to uniquely quantify the localization of lung phagocytes. Although IHC faces limitations in terms of diversity of fluorophores that can be used for cell identification compared to flow cytometry, the use of selected surface or intra-cellularly expressed markers in combination with cellular localization allowed the identification of all major phagocyte subsets. Not unexpectedly, AMs were found exclusively in the alveolar lumen, located adjacent to epithelial cells, as previously observed (2, 6, 23). Consistent with the study by Gibbings et al., IM subpopulations clustered in the bronchial interstitium and around blood vessels (5). While our study does contrast with the study of Bedoret et al. who observed CD11c^−^CD11b^+^MHC-II^+^ cells only in the alveolar interstitium, the studies are concordant in the observation of interactions between IMs and DCs (7). In contrast to previous reports describing cDC localization in the alveolar interstitium (6, 24, 25), we observed a clear accumulation of both CD11b^+^ and CD103^+^ DCs in the connective tissue between airways and pulmonary blood vessels (summarized in Figure 9). However, previous studies did not identify vessels within the lung, suggesting that cDCs may have indeed been present next to small vessels within the alveolar interstitium (6, 24, 25). Our observation of cDCs in tissues between airways and pulmonary vessels is also in accordance with the observation that T lymphocytes are clustered around arteries and veins that accompany airways in a region that harbors also lymph vessels (26) and suggests a locale for pulmonary DC:T-cell contacts further strengthening the hypothesis of an immunologically important microenvironment between airways and arteries as well as veins. Interestingly, we observed no difference in cellular distribution of CD11b^+^ and CD103^+^ DCs, although CD103^+^ DCs were originally considered as intraepithelial cells sampling antigen from the airway lumen (27–30). Therefore, our data, as recently published studies of Veres et al. and Thornton et al., raise the question whether the reported intraepithelial localization might not be limited to a minor fraction of CD103^+^ cDCs (24, 31).

Antigen uptake and processing is a key function shared by phagocytic cells, regardless of subset, but differences in relative ability to take up and process antigen have also been proposed to determine overall capacity to induce pro-inflammatory versus tolerogenic responses. Our study revealed that IM1 followed by IM2 and CD11b^+^ DCs were the most accomplished phagocytic cells. Limited antigen uptake was observed in CD103^+^ cDCs. These observations are somewhat consistent with those of Gibbings et al., which demonstrated preferential antigen uptake of latex beads or zymosan-coated particles (but no *Escherichia coli*-derived particles) by IM1 following *in vivo* exposure (5). Why IM1 are better able to phagocytose particles remains unclear. It is unlikely to be related to excess allergen levels as similar results were observed in our *in vitro* and *in vivo* setting, and it is likely due to true differences in phagocytic capacity (rather than differences in antigen processing/lysosomal acidification) as similar results were observed with OVA-Alexa Fluor (AF) 647, which emits fluorescence in the absence of antigen processing (data not shown). These differences may be due to differences in preferred antigen between IMs and CD11b^+^ DCs or lower protease expression, as has been reported elsewhere (32, 33). Finally, it is also interesting to note that allergen uptake appeared independently of the distance to airways and distant from the alveoli, suggesting that uptake was not driven exclusively by the existence of epithelium-spanning DC protrusions as has been observed by Thornton et al. (24). This study described that antigen uptake occurs via processes of CD11b^+^DCs that penetrate the epithelial layer and thereby direct contact between CD11b^+^ DCs and antigens in the alveolar lumen or the airway. These CD11b^+^ DCs likely match moDCs/IM3. Similar mechanisms were previously shown in studies performed in a model of the human epithelial airway wall or the gut (34, 35). In contrast, our study did not provide any indications that processes of CD11b^+^ DCs directly reach the alveolar lumen by penetrating the epithelial layer with cell-spanning processes. Instead, we observed antigen uptake also in regions more distant from the alveolar lumen, indicating mechanisms of antigen uptake independent of protrusions through the epithelial layer. Altogether, our data confirm other studies which reported no evidence of such epithelial cell-spanning processes (29, 31) despite claims to the contrary oft-reported in review articles (36–38). Thus, the observations of Thornton et al. remain unclear in naive mice (24).

One of the more exciting findings of the current study is the discovery of specific contacts between IM2 and CD103^+^ cDCs around airways and blood vessels in the naive lung, and a decrease in the frequency of these contacts 24 h after antigen encounter. Interestingly, in the gut, a comparable interaction between CD103^+^ DCs and CX_3_CR1^+^ monocyte-derived macrophages is crucial for tolerance to food allergens (17). These interactions were described to induce oral tolerance by enabling the transfer of oral antigens from CX_3_CR1 macrophages with high uptake efficiency to Treg-inducing CD103^+^ DCs via Connexin43 (Cx43) channels. Our data indicate a comparable mechanism in the lung that enables exchange of inhaled antigen from antigen-uptaking IM2 to less phagocytic CD103^+^ DCs. Preliminary data from our group indicate that also pulmonary CD103^+^ DCs express Cx43 (data not shown), but the expression on IM2 still has to be proven. However, the expression of Cx43 in the lung has already been published for AMs and pulmonary epithelial cells. Therefore, Cx43-mediated communication between AMs, using epithelial cells as a conduit for synchronization of “waves” of Ca2^+^, represents an important anti-inflammatory signal (4). Moreover, interactions between IMs and pulmonary DCs (but not AMs and DCs) were found to decrease the maturation and migration of DCs loaded with a normally tolerogenic allergen in an IL-10-dependent manner (7). As macrophage/DC interactions are typically tolerogenic (4, 7, 17), it is tempting to speculate that their observed reduction following administration of a potent aeroallergen (HDM) may represent a fundamental mechanism whereby pulmonary immune responses can be initiated—by specific abrogation of cell–cell contacts linked with the development of tolerance. However, at this point, we cannot exclude the possibility that the apparent down-regulation of IM2:CD103^+^ cDC contacts represents a modulation of IM2-defining cell surface markers (e.g., CD11b^+^). This is an area of active investigation in our lab. Furthermore, our study allows yet only snap-shot analyses on cell localizations and cellular interactions but does not enable conclusions on spatiotemporal changes of phagocyte subsets in a living organ. However, information about duration and quality of cell interactions is critically necessary and will be, therefore, addressed in further studies in our lab using viable thick lung slices in an oxygenated microscope chamber as well as FACS isolation of distinct phagocyte subsets and subsequent PCR analysis of functional markers.

In summary, our study established a comprehensive picture of lung phagocytes by IHC. This directly enabled us to determine differential functional aspects of the identified subpopulations as well as cellular contacts between subpopulations. We are of the opinion that these observations will help to direct future research concerning the cellular interplay during allergic sensitization efficiently.

## Materials and Methods

### Mice

C57BL/6 mice (Charles River Laboratories) were maintained in the University of Lübeck specific pathogen-free facility and used at 8–12 weeks of age. Animal care was provided in accordance with German rights. These studies were reviewed and approved by the Schleswig-Holstein state authorities [No. V242-7224.122-1 (39-2/13)].

### Allergic Sensitization *In Vivo*

C57BL/6 mice were immunized intratracheally with 100 μg/50 μL of HDM (crude extract; Greer Laboratories). Alternatively, mice were immunized intratracheally with a mixture of 40 µg DQ-OVA (Life Technologies) and 100 µg HDM in 50 µL PBS (Gibco Life Technologies). Mice were sacrificed after 4 or 24 h, and precision cut lung slices (PCLS) were prepared and stained for further microscopic analysis.

### Lung Explantation and Preparation of PCLS

PCLS were prepared as described earlier (26). Briefly, mice were anesthetized by inhalation of isoflurane (Baxter) and the trachea was exposed. After lung perfusion (4.7 mL HEPES-buffered Ringer solution and 0.3 mL heparin sodium solution) the lung was filled with agarose (Bio-Rad) via the trachea and removed from the thorax. The left lung was fixed on an aluminum block and cut into 300 µm thick slides. Alternatively, the lung of DQ-OVA/HDM-treated animals was fixed before removal with 1% paraformaldehyde (Merck) in PBS for 20 min with two subsequent washing steps with PBS (38.5 mM NaCl (C. Roth), 0.425 mM Na_2_HPO_4_·H_2_O (Merck), 2 mM Na_2_HPO_4_·10H_2_O (Merck) and further prepared as described before (26).

### Analysis of Lung Structures by IHC and Autofluorescence

Airway and blood vessels were identified by using a primary antibody anti-α-SMA-Cyanin (Cy) 3 (1A4; Sigma-Aldrich) staining smooth muscle cells of airways and vessels or using autofluorescence (excitation: 488 nm; detection: 490–530 nm).

### Direct and Indirect IHC

PCLS were defrosted at RT on a shaker and washed three times in 1 mL TBS [7.43 mM Tris, 43.5 mM Tris-HCl, 150 mM NaCl (all C. Roth)] for 10 min. If unlabeled antibodies were used, slices were incubated with 5% normal donkey serum (Linaris) in 500 µL of TBS for 30 min to block unspecific binding sites of the secondary antibody. Blocking was not necessary for stainings with directly labeled antibodies. Primary antibodies were diluted in TBS and incubated overnight at RT. Slices were rinsed three times in TBS for 10 min. If no secondary antibody was needed, PCLS were transferred to a slide, dried carefully, and coverslipped with Mowiol mounting medium [12 g Mowiol 4-88 (Hoechst), 30 mL A. bidest., 60 mL Tris buffer (0.2 M), 30 g glycerine (pH 8.5) (Merck)]. Alternatively, PCLS were incubated with secondary antibodies for 60 min. Slices were rinsed three times with TBS minimum and coverslipped as described above. The following antibodies were used to identify phagocyte subpopulations: anti-CD11c-AF488 (clone N418) (eBiosciences), anti-CD11b-Brilliant Violet (BV) 421 (clone M1/70), anti-mPDCA-1-Phycoerythrin (PE) (clone 927), anti-MHC-II-PE (clone M5/114.15.2), anti-CD103-PE (clone 2E7) (all BioLegend), purified anti-Langerin (polyclonal E17) (Santa Cruz), and anti-CD64-PE und AF647 (clone X54-5/7.1) (BD Biosciences). As secondary antibody donkey anti-goat IgG-AF488, donkey anti-goat IgG-AF647, donkey anti-goat IgG-Cy3, and donkey anti-rat IgG-Cy3 (all Invitrogen) were used.

### *In Vitro* Antigen Stimulation

Viable lung slices were incubated at room temperature with a mixture of 25 µg DQ-OVA and 30 µg HDM diluted in 500 µL PBS in 24-well plates on a shaker for 30 min. Afterward, the slices were fixed with 1% PFA at room temperature for 30 min and immunohistochemically stained as described previously for microscopic analysis.

### Confocal Microscopy

Stained slices were evaluated with confocal microscopy. Z-stacks (40 µm with a resolution of 1 µm) were recorded to allow a three-dimensional presentation of lung structures including possible cell interactions. Analysis was performed using a FV1000 (Olympus), a LSM 510Meta (Zeiss), and a LSM 710 (Zeiss) confocal microscope.

### Quantification of Lung Phagocytes

For total cell quantification, PCLS were prepared and stained as described above. Weight was assessed before mounting. The thickness of the slice was determined by microscopy. The area of the slice was determined by hand. Areas of image acquisition were distributed randomly by blind relocation of the slice. Images were acquired using a confocal microscope [FV1000 (Olympus) and LSM 710 (Zeiss)] with specified parameters (see below) using a 20× immersion objective. During image analysis, cells were counted manually and assigned by their surface marker expression using the Imaris software (Version7.2) (Bitplane). The cell number per slice was determined by the ratio counted cells:area. The cell number per lobe was calculated by the weight ratio slice:lobe. Total cell number was determined by the weight ratio left lung:total lung.

The calculation of the total cell number (x) was done using the following formula:

$$x=a\times \left(\frac{e}{c\times b}\right)\times \left(\frac{f}{d}\right)\times \left(\frac{h}{g}\right)\times \left(i\right)$$

*a* = counted cell numbers; *b* = number of images; *c* = area (image) = 636 µm × 636 µm; *d* = thickness of the stack = 40 µm; *e* = area (slice); *f* = measured thickness of the slice; *g* = weight of the slice; *h* = weight of the lobe; *i* = weight ratio left lung to total lung; *x* = cell number.

### Analysis of Antigen Uptake by Confocal Microscopy

Analysis of antigen uptake was performed on a LSM 710 confocal microscope (Carl Zeiss) using multiple tracks. Z-stacks were detected for a range of 5 µm using a resolution of 1 µm. DQ-OVA fluorescence was measured in the FITC-Channel. The phagocyte subtypes were identified by staining with purified anti-CD11c (clone N418), anti-CD11b-BV421 (clone M1/70), and anti-MHC-II-AF647 (clone M5/114.15.2) (Biolegend) antibodies. As secondary antibody, goat anti-armenian hamster IgG-Cy3 (Invitrogen) was used. For the quantification of OVA uptake, phagocytes of at least six images of randomly distributed airways and adherent arteries per mouse were analyzed for OVA co-localization using Imaris.

### Lung Extraction for Flow Cytometry and Cell Isolation

Bronchoalveolar lavage (BAL) was performed by cannulating the trachea, injecting 1 mL of PBS and subsequently aspirating the BAL fluid. Liberase/DNAse I (both Sigma-Aldrich) digests of the lung were prepared to obtain single lung cell suspensions (21). Single lung cell suspensions were used for the analysis of phagocyte subpopulations by flow cytometry.

### Staining for Flow Cytometry

Single cell suspensions were blocked for unspecific binding with anti-CD16/32 (eBiosciences). Subsequent staining with anti-SiglecF-BV421 (E50-2440) (all BD Biosciences), anti-CD11c-Allophycocyanin (APC) (N418), anti-CD3e-eFluor (eF) 450 (145-2C11), anti-CD49b-eF450 (DX5), anti-CD19-eF450 (1D3) (all eBiosciences), anti-CD11b-BV510 (M1/70), anti-CD103-Peridin chlorophyll protein Cyanin (PerCP-Cy) 5.5 (2E7), anti-MHC-II-APC-eF780 (M5/144.15.2), anti-CD64-PE (X64-5/7.1) (all Biolegend), and anti-C5aR1-PE (20/70, AbD serotec) was performed and subsequently analyzed using a BD Aria II (BD Biosciences).

### Statistical Analysis

Statistical analysis was performed using the GraphPad Prism version 5 (GraphPad Software, Inc.). Data are represented as mean and SEM. Statistical differences were evaluated by Mann–Whitney *U*-test, Kruskal–Wallis test, or two-way ANOVA. *p* values <0.05 were considered statistically significant.

## Ethics Statement

These studies were reviewed and approved by the Schleswig-Holstein state authorities (Nr. V242-7224.122-1 (39-2/13)).

## Author Contributions

FH, JB, YL, IPL, IS, and PK contributed to the conception and design of the study. FH, JB, and IL performed experiments. FH, JB, IL, IS, and PK analyzed the data. IS wrote the first draft of the manuscript. All authors contributed to manuscript revision, read and approved the submitted version.

## Conflict of Interest Statement

The authors declare that the research was conducted in the absence of any commercial or financial relationships that could be construed as a potential conflict of interest.
