# Supplementary material for: Distribution and Interaction of Murine Pulmonary Phagocytes in the Naive and Allergic Lung
Source: Front Immunol. 2018 May 16;9:1046. doi: 10.3389/fimmu.2018.01046 (PMC5964136; doi:10.3389/fimmu.2018.01046)
Supplement: Supplementary file 1 [file Presentation_1.PDF]

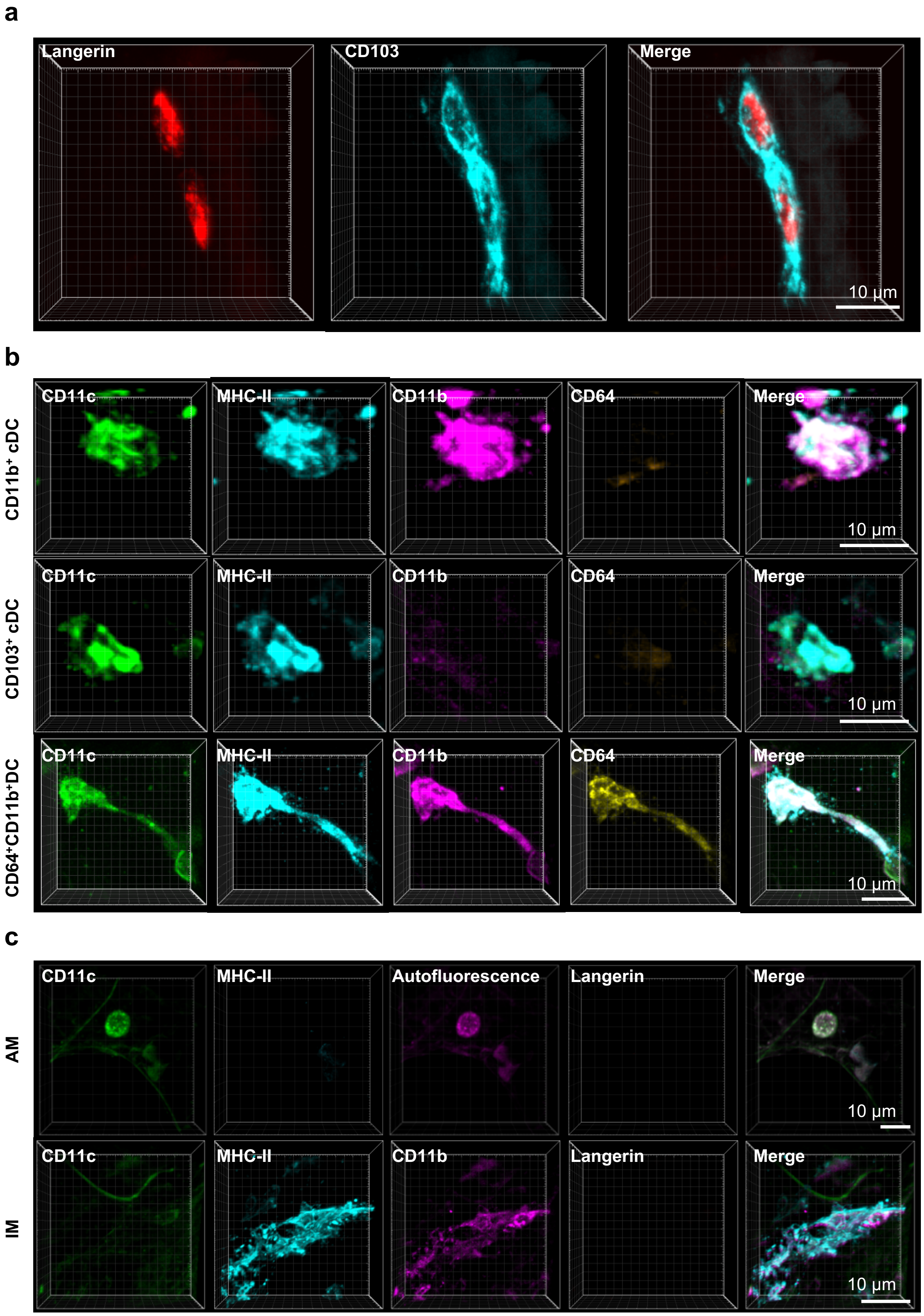

Suppl. Figure 1: Control staining of Lung phagocyte subsets. Precision cut lung slices (PCLS) (300 µm) from naïve C57BL/6 mice were generated. **(a)** Slices were stained with anti-Langerin and anti-CD103 ABs. Stained slices were evaluated with confocal microscopy. Single-color and merged color display with Langerin (red) and CD103 (turquois). **(b, c)** Slices were stained with anti-CD11c, anti-MHC-II, anti-CD11b and anti-Langerin or anti-CD64 ABs. Stained slices were evaluated with confocal microscopy to identify CD11b$^+$ dendritic cells (DCs), CD103$^+$ DCs and CD64$^+$CD11b$^+$ DCs **(b)** or alveolar macrophages (AMs) and interstitial macrophages (IMs) **(c)**. Single-color and merged color display with CD11c (green), MHC-II (turquois), CD11b or autofluorescence (purple) and Langerin (red) or CD64 (yellow). Data are representative of at least three independent experiments.

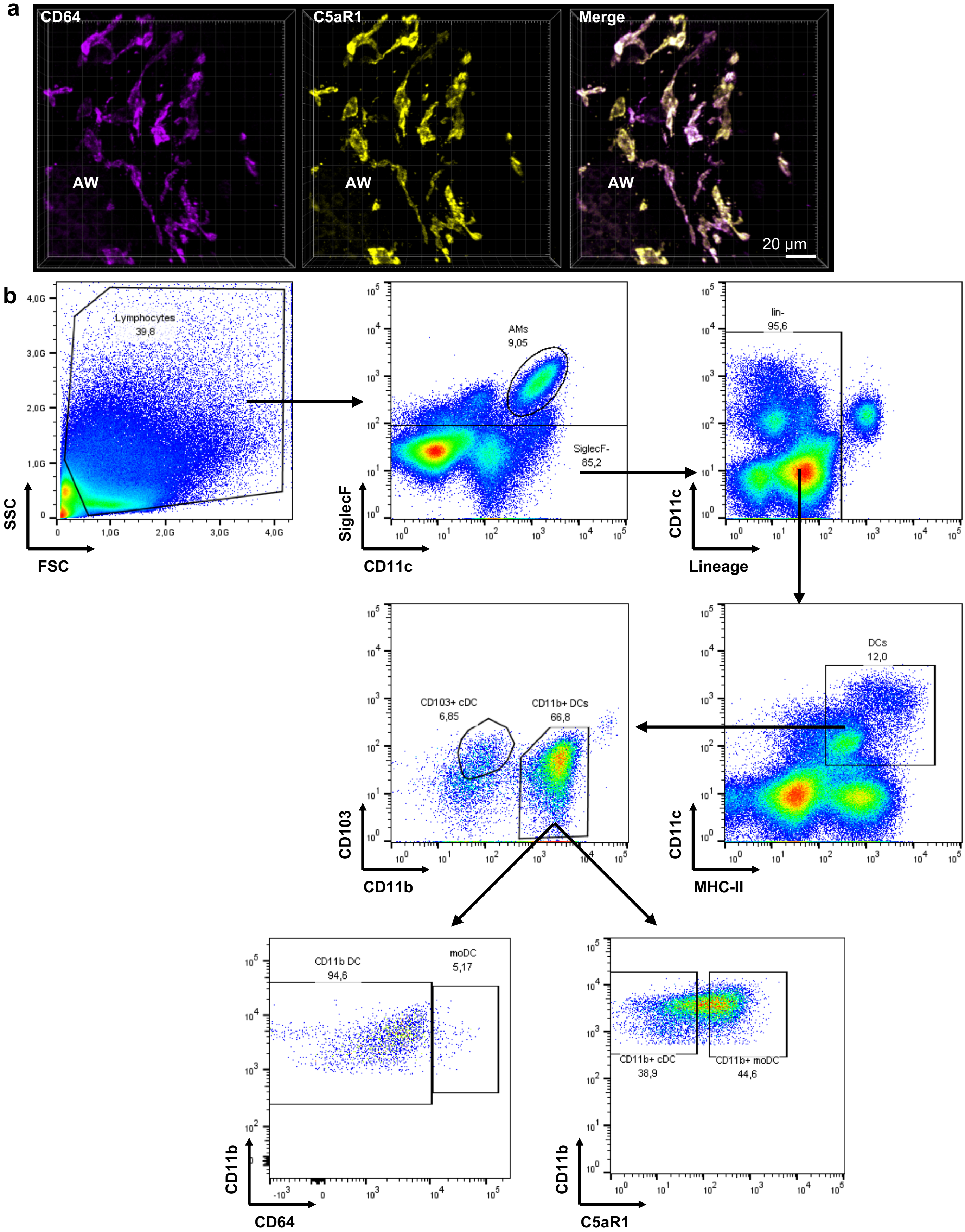

Suppl. Figure 2: Expression of C5aR1 and CD64 in lung tissue by IHC and flow cytometry. (a) Precision cut lung slices (PCLS) (300 µm) from naïve C57BL/6 mice were generated and stained with anti-CD64 and anti-C5aR1 mABs. Stained slices were evaluated with confocal microscopy. Single-color and merged color display with CD64 (purple) and C5aR1 (yellow). Data are representative of at least four independent experiments. AW: airway. **(b)** Single cell suspensions from lungs of naïve C57/BL6 mice were generated and stained for flow cytometric analysis. In a first step cellular debris was excluded from further analyses (left panel, top). Eosinophils and alveolar macrophages (AMs) were excluded from further analyses based on the expression of SiglecF and CD11c (middle panel, top). Among the remaining cells B cells, T cells, NK cells and neutrophils were excluded by staining of CD3e, CD19, CD49b and Ly6G (last panel, top). Dendritic cells (DCs) were identified based on the expression of CD11c and MHC-II (last panel, middle). CD11b^{+} and CD103^{+} DCs were identified (middle panel, middle). CD11b^{+} DCs were analyzed for the expression of CD64 (left, bottom) or C5aR (right, bottom). Data are representative of a pool of 30 naïve C57/BL6 mice.

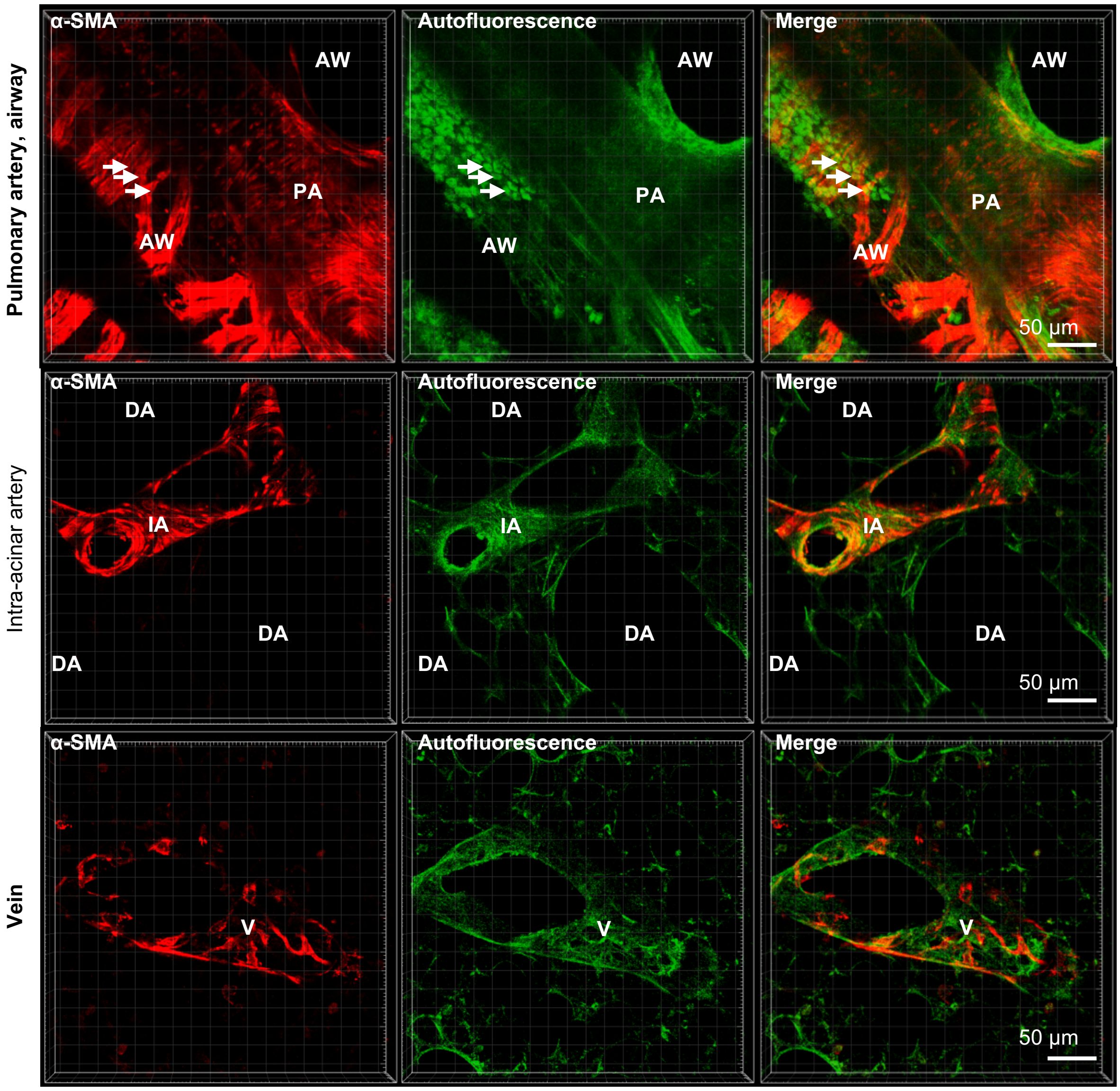

Suppl. Figure 3: Visualization of airway and blood vessels based on antibody staining or autofluorescence. Precision cut lung slices (PCLS) (300 µm) from naïve C57BL/6 mice were generated and stained with anti-alpha-smooth muscle actin (α-SMA) mAB. Stained slices were evaluated with confocal microscopy. Single-color and merged color display with α-SMA (red) and autofluorescence (green). Autofluorescence was excited at 488 nm and recorded at wavelengths between 490-530 nm. Data are representative of at least two independent experiments. AW: airway; DA: alveolar duct; PA: pulmonary artery; IA: intra-acinar artery; V: vein.

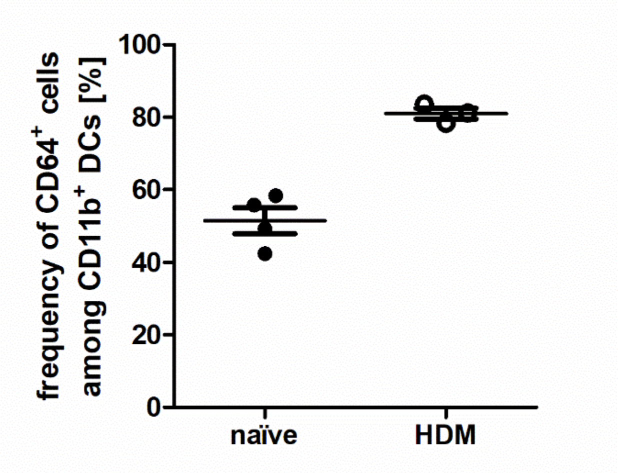

Suppl. Figure 4: Allergic sensitization induces cellular accumulation of $CD64^{+}CD11b^{+}$ DCs. C57BL/6 mice were anaesthetized and immunized intratracheally with house dust mite extract (HDM) (100 µg). 24 hours after immunization mice were sacrificed, precision cut lung slices (PCLS) (300 µm) were generated and stained with anti-CD11c, anti-MHC-II, anti-CD11b and anti-CD64 mABs. Naïve C57/BL6 mice served as controls. Stained slices were evaluated with confocal microscopy. The frequency of $CD64^{+}CD11b^{+}$ DCs was determined. Lines indicate mean ± SEM.
